# Parkinson’s Disease in Kazakhstan: Clinico-Demographic Description of a Large Cohort

**DOI:** 10.3233/JPD-191782

**Published:** 2020-04-03

**Authors:** Rauan Kaiyrzhanov, Nazira Zharkinbekova, Chingiz Shashkin, Talgat Khaibullin, Gulnaz Kaishibayeva, Vadim Akhmetzhanov, Dinara Z. Sadykova, Zhanar Seidinova, Anjela Taskinbayeva, Altynay Karimova, Mie Rizig, Henry Houlden

**Affiliations:** a University College London, Institute of Neurology, Department of Neuromuscular Disorders, Queen Square, London, UK; b South Kazakhstan Medical Academy, Department of Neurology, Shymkent, Kazakhstan; c Semey Medical University, Department of Neurology, Semey, Kazakhstan; d Institute of Neurology Named After S.K. Kaishibayev, Almaty, Kazakhstan; e Astana Medical University, Nur-Sultan, Kazakhstan

**Keywords:** Parkinson’s disease, Parkinson’s disease in Asia, Central Asia, age of onset, progression

Little is known about Parkinson’s disease (PD) epidemiology, demographics, genetics and clinical presentation in Central Asian countries. Western and East Asian studies have suggested that PD might have variable clinico-demographic characteristics in different ethnic groups [1–3]. In this regard, here we make a first demographic and clinical analysis of a large PD cohort in Kazakhstan, the biggest Central Asian country, residing between Europe and East Asia.

We conducted a retrospective study reviewing outpatient clinical records of PD patients from the three medical centers across the country with an available movement disorders specialist. Only cases compatible with UK Brain Bank PD diagnosis criteria [4] were selected. Available clinico-demographic data was systematically retrieved from the records. Patients were defined as early-onset PD (EOPD) if the disease started before the age of 50 [5].

The clinical records of 990 patients with PD diagnosis were eligible for the analysis. The mean age of PD onset was 55.01±9.7 years (range, 14-82) and the mean age at examination was 62.2±9.0 years (range, 28–86). Almost half of the cases (43.5%) were between the ages of 61–70 at the moment of the last examination. Mean disease duration was 7.1±5.2 years (range, 0-38). (The results are presented in Supplementary Table 1 and Supplementary Figure 1).

EOPD cases composed 30.5% in our cohort, and 7.6% developed PD before the age of 40 years. The male to female (M: F) ratio among the whole cohort and young-onset PD cases was 1 : 1.3 and 1 : 0.99, respectively. The prevalence of females has been noticed in our PD cohort. The mean age of onset was significantly younger in males compared to females (53.9 vs 55.8, *p* = 0.002, corrected for multiple comparisons). The mean age at an examination in males was also significantly lower than in females (61.2±9.5 vs 63.0±8.5, *p* = 0.003, corrected for multiple comparisons). Mean disease duration did not differ between genders (*p* = 0.4). An autosomal dominant family history of PD was found in 4% of patients being more prevalent among EOPD.

The most prevalent PD subtype was akinetic-rigid. The Mean Hoehn-Yahr stage (HYS) was 2.3±0.8 and it did not differ between genders (*p* = 0.16). Only 207 (23.7%) patients had an available off stage MDS UPDRS motor score with a mean 34.9±18.2 (range, 2-100). EOPD cases had significantly higher MDS UPDRS motor scores and HYS compared to late-onset PD (LOPD) patients (39.5±19.4 vs 32.8±17.2, *p* = 0.02; 2.5±0.7 vs 2.2±0.8, *p* < 0.01, corrected for multiple comparisons). UPDRS motor score did not differ between genders (males 33.8±17.4 vs female 35.3±17, *p* = 0.5, corrected for multiple comparisons). Dyskinesias were expressed by 17.1% of patients, and 14.5% had both dyskinesias and motor fluctuations. Female and EOPD subjects expressed slightly more levodopa-induced complications.

Almost 68% of patients were on levodopatherapy with a median daily dose of 500 mg (range, 250-2500 mg, in 42% of patients 400 mg/day, in 58% 500 mg/day). Thirty-four percent of patients were on levodopa monotherapy only. In terms of other anti-parkinsonian medications, 9.8% were taking amantadine, 16.1% were taking dopamine agonists (DA), 8.1% were on rasagiline, and 6% were on trihexyphenidyl.

The mean age at onset (AAO) in our study (55.01) was younger than in Caucasian (58.0±12) and slightly older than in East Asian (54.0±12) PD patients [6]. The relatively young age of PD onset in the Kazakhstani cohort might be influenced by unexplored population-specific genetic factors, environmental agents, the character of diet, and comorbidity.

PD tends to affect males more than females in Western countries, and the opposite has been observed in East Asian countries. [7-10]. Similar to East Asia, female cases dominated in our whole cohort, however among young-onset cases the significant difference between genders has not been seen. Interestingly, a comprehensive meta-analysis of age-adjusted M: F incidence ratios for PD by Taylor et al. [11] concluded that the observed gender difference is not the same across the age groups, and the male predominance in PD in Western populations possibly restricted to LOPD cases.

Male subjects developed PD younger in comparison to females in our study. Although AAO of PD in female subjects was older, mean MDS UPDRS motor scores did not differ between genders. This, bearing in mind the equal mean disease durations in both genders in our study, could suggest similar rates of the disease progression between genders.

Less than one in five PD patients in our cohort expressed levodopa induced-dyskinesias and/or motor fluctuations. Considering the mean disease duration (7.1±5.2) and the daily dose of levodopa of 500 mg and more in 58% of patients, one would expect higher rates of motor complications. Interestingly, low rates of dyskinesias and motor fluctuations have also been reported in Western Pacific countries [2]. Deeper knowledge in inter-ethnic genetic variations in postsynaptic dopamine maintenance could shed light on the biological basis of the observed low rates of levodopa complications in Asians [2]. Important to note, that mild levodopa complications, especially motor fluctuations, could have been under-acknowledged by movement disorders specialists in Kazakhstan, and were not mentioned in the clinical records that this study reviewed.

The analysis of antiparkinsonian medications showed that the main PD medication in Kazakhstan is levodopa, which was taken as monotherapy in one-third of patients. The low proportion of consumed DA and monoamine oxidase inhibitors could suggest either limited access due to the high cost of these medications in the country or that the importance of adjunct levodopa therapy is under-recognized by local neurologists who follow-up PD patients.

The retrospective nature of our study appears to be the major limitation. Data were assembled from clinical records, and therefore not every case had available UPDRS scores, detailed information about family history, non-motor symptoms, and mild levodopa-induced complications.

Considering all the findings and limitations of this study together, one might infer that well- designed high-quality epidemiological, genetic, and observational studies are warranted in Kazakhstan and other Central Asian countries to fill the gap in the knowledge of PD in the extensive and multinational region.

## CONFLICT OF INTEREST

The authors have no conflict of interest to report.

## Supplementary Material

Supplementary Material
