# Supplementary material for: Parkinson’s Disease in Kazakhstan: Clinico-Demographic Description of a Large Cohort
Source: J Parkinsons Dis. 2020 Apr 3;10(2):707–9. doi: 10.3233/JPD-191782 (PMC7242829; doi:10.3233/JPD-191782)
Supplement: Supplementary Material [file jpd-10-jpd191782-s001.pdf]

Supplementary Material

Parkinson's Disease in Kazakhstan: Clinico-Demographic Description of a Large Cohort

Supplementary Table 1. The demographic and clinical profile of the cohort in this study

	All cohort	Males	Females	EOPD	LOPD
**Mean age of onset, (y)**	55.0 ± 9.7	53.9 ± 8.2	55.8 ± 7.3		
		p=0.002			
**Mean age at examination, (y)**	62.2 ± 9.0	61.2 ± 9.5	63.0 ± 8.5		
		p=0.003			
**Mean disease duration, (y)**	7.1 ± 5.2	7.2 ± 17.1	10.8 ± 7.1		
		p=0.4			
**Mean UPDRS motor score**	34.9 ± 18.2	33.8 ± 17.4	35.3 ± 17	39.5 ± 19.4	32.8 ± 17.2
		p=0.5		p=0.02	
**Mean HYS**	2.3 ± 0.8	2.2 ± 0.8	2.3 ± 0.8	2.5±0.7	2.2±0.8
		p=0.16		p<0.01	
**Onset before 50 years old, % (n)**	30.5% (302)	36% (152)	27% (150)		
**Onset before 40 years old, % (n)**	7.6% (76)	9.9% (42)	5.7% (32)		
**M:F ratio, (total number)**	1:1.3 (M-426, F- 564)			1:0.99 (M-152, F-150)	1:1.5 (M-274, F-414)
**FH, % (n)**	4% (40)	4.7% (20)	3.5% (20)	7.6% (23)	2.3% (16)
**Dyskinesia, % (n)**	17.1% (170)	14.6% (62)	19.1% (108)	30.1% (91)	11.4% (79)
**Dyskinesia plus fluctuations, % (n)**	14.5% (144)	11.7% (50)	16.7% (94)	27.1% (82)	9% (62)
**Median dose of levodopa, mg (range, mg)**	500 (250-2500)			500	500
**On levodopatherapy, % (n)**	68% (673)				
**Levodopa monotherapy, % (n)**	34% (336)				
**On Amantadine, % (n)**	9.8% (97)				
**On DA, % (n)**	16.1% (159)				
**On Rasagiline, % (n)**	8.1% (80)				
**On trihexyphenidyl, % (n)**	6% (60)				

UPDRS, Unified Parkinson's disease rating scale; HYS, Hoehn-Yahr stage; M:F ratio, male to female ratio; FH, family history; PD, Parkinson's disease; LOPD, late-onset Parkinson's disease; YOPD, early-onset Parkinson's disease, DA, dopamine agonists

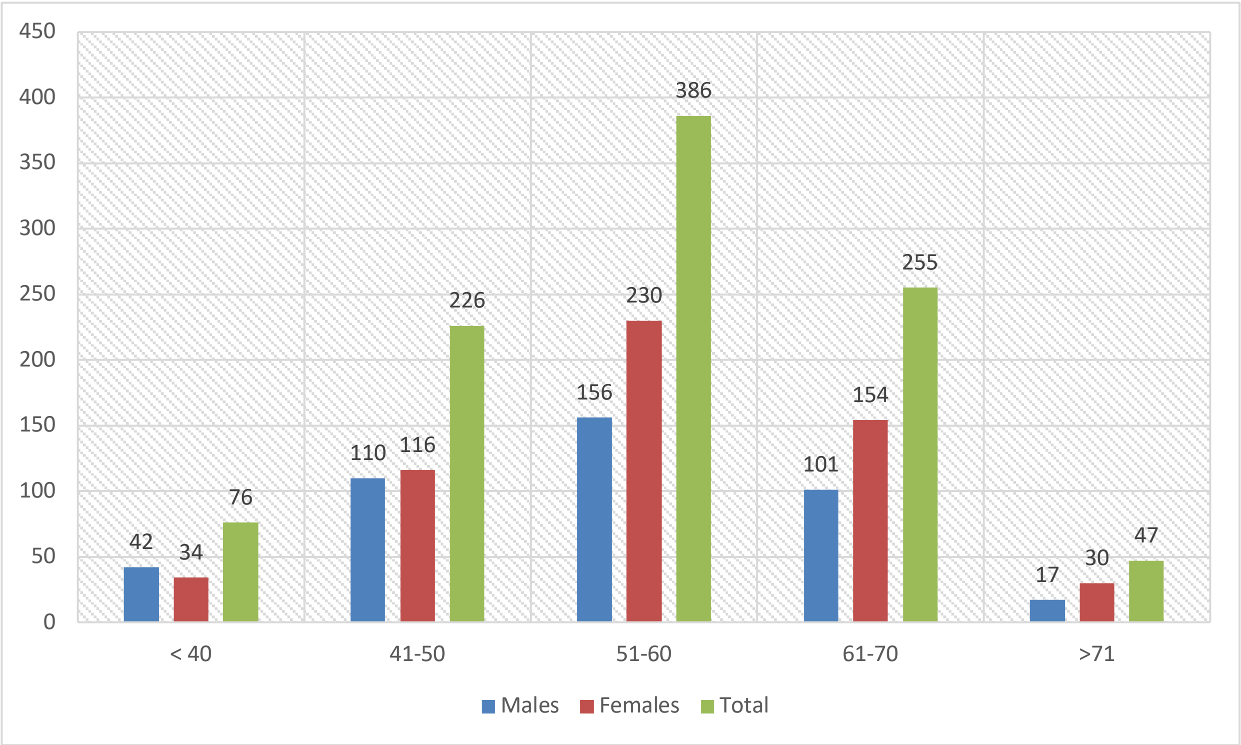

Supplementary Figure 1. The gender distribution for Parkinson's disease onset in the age of decades. The figure shows that the high pick for Parkinson's disease onset for both genders in this study was between 51-60 years old. Vertical axis – the absolute number of patients, horizontal axis – decades for age.
